# Diagnosis of Syphilis by Laboratory Methods

**Published:** 1915-10

**Authors:** Henry H. Morton

**Affiliations:** Brooklyn, N.Y.


**Diagnosis of Syphilis by Laboratory Methods,** by Henry H. Morton, M. D., Brooklyn, N. Y.

1. The demonstration of the spirocheta pallida in the suspected lesion.
2. The Wasserman reaction in the blood.
3. The cutaneous or Luetin reaction of Noguchi.
4. The findings in the spinal fluid.

Clinical experience must supplement laboratory diagnosis, for sometimes laboratory reports are misleading.

For the demonstration of the spirocheta the "Dark Field" is the best method as the living spirocheta may be seen. The India ink and Giemso stains are also extensively used for the demonstration of spirocheta in smears, while the Levaditi stain is used to demonstrate the spirocheta in tissues.

The Wasserman reaction is depended upon for the diagnosis of Syphilis today. The old method is the best, as short cuts are unreliable. A positive reaction appears 6 to 8 weeks after infection. 100 per cent of secondary syphitics untreated give a positive reaction. In some cases, in spite of good treatment, it is impossible to change the reaction, if, however, it is reduced but is not negative it shows the need of more treatment. In suspected cases where the Wasserman is negative a small dose of Salvarsan or Mercury will often cause the reaction to be positive in 24 to 48 hours. This is called a provocative Wasserman.

The Luetin reaction was devised by Noguchi in 1911. A mixed culture of killed spirochetae are injected beneath the skin. A positive reaction as a papule or pustle appears in 3 to 5 days. Nichols and Craig found that the positive reaction appeared more consistently in latent cases.

The findings in the spinal fluid are useful in diagnosing cerebro-spinal syphilis. There is very little danger in lumbar puncture if properly done. In examining the spinal fluid we estimate the pressure, make a cell count, estimate the globulin and do a Wasserman. Up to 5 cells per cm. is normal, 5 to 10 is doubtful, and over 10 is pathological. Increased cell count shows irritation along the cord. Globulin is not normally found in spinal fluid, and its presence shows irritation. It is present in 80 to 100 per cent. of syphitic nervous disease.

In syphilitic nervous disease the Wasserman reaction is usually present. In Tabes about 60 to 70 per cent. positive. In paresis 100 per cent positive.

